# Neoamphimedine Circumvents Metnase-Enhanced DNA Topoisomerase IIα Activity Through ATP-Competitive Inhibition

**DOI:** 10.3390/md9112397

**Published:** 2011-11-18

**Authors:** Jessica Ponder, Byong Hoon Yoo, Adedoyin D. Abraham, Qun Li, Amanda K. Ashley, Courtney L. Amerin, Qiong Zhou, Brian G. Reid, Philip Reigan, Robert Hromas, Jac A. Nickoloff, Daniel V. LaBarbera

**Affiliations:** 1 Department of Pharmaceutical Sciences, The University of Colorado Skaggs School of Pharmacy and Pharmaceutical Sciences, Anschutz Medical Campus, Aurora, CO 80045, USA; E-Mails: jessica.ponder@ucdenver.edu (J.P.); byong.yoo@ucdenver.edu (B.H.Y.); adedoyin.abraham@ucdenver.edu (A.D.A.); qun.li@ucdenver.edu (Q.L.); qiong.zhou@ucdenver.edu (Q.Z.); brian.reid@ucdenver.edu (B.G.R.); phillip.reigan@ucdenver.edu (P.R.); 2 Department of Environmental and Radiological Health Sciences, Colorado State University, Fort Collins, CO 80523, USA; E-Mails: ashleyak@ad.nmsu.edu (A.K.A.); camerin@rams.colostate.edu (C.L.A.); j.nickoloff@colostate.edu (J.A.N.); 3 Department of Medicine, University of Florida and Shands Health Care System, Gainesville, FL 32610, USA; E-Mail: Robert.hromas@medicine.ufl.edu

**Keywords:** neoamphimedine, topoisomerase II, Metnase, cancer therapeutics

## Abstract

Type IIα DNA topoisomerase (TopoIIα) is among the most important clinical drug targets for the treatment of cancer. Recently, the DNA repair protein Metnase was shown to enhance TopoIIα activity and increase resistance to TopoIIα poisons. Using *in vitro* DNA decatenation assays we show that neoamphimedine potently inhibits TopoIIα-dependent DNA decatenation in the presence of Metnase. Cell proliferation assays demonstrate that neoamphimedine can inhibit Metnase-enhanced cell growth with an IC_50_ of 0.5 μM. Additionally, we find that the apparent K_m_ of TopoIIα for ATP increases linearly with higher concentrations of neoamphimedine, indicating ATP-competitive inhibition, which is substantiated by molecular modeling. These findings support the continued development of neoamphimedine as an anticancer agent, particularly in solid tumors that over-express Metnase.

## 1. Introduction

Type IIα DNA topoisomerase (TopoIIα) plays a critical role in cell cycle progression by relieving various types of topological strain induced in DNA double strands during replication, transcription, and chromosome segregation, making it a crucial target for chemotherapy in several cancers [1–3]. The clamp-shaped nuclear homodimer causes transitory double-stranded breaks in DNA, allowing it to pass a second double strand through before re-ligating the broken strand, fueled by ATP hydrolysis[4]. This process generates an intermediate complex in which TopoIIα is covalently bound to the cleaved DNA, known as the cleaved complex [3,5]. Conventional TopoIIα poisons, such as doxorubicin and etoposide, act by stabilizing this complex, leading to its accumulation, DNA lesions, and ultimately, apoptosis. However, accumulation of this cleaved complex can lead to chromosomal translocations contributing to adverse side effects including secondary malignancies [2]. One of the exclusive functions of TopoIIα is to decatenate (disentangle) sister chromatids before segregation [5]. Metnase, a recently identified double-strand break repair factor, interacts with TopoIIα and this interaction has been shown to enhance TopoIIα-dependent DNA decatenation [6]. Recent studies of this interaction show that Metnase can promote tumor cell growth in the presence of conventional TopoIIα poisons, which may be an important factor in multidrug resistance (MDR) in malignant cancers over-expressing Metnase [7,8]. Therefore TopoIIα inhibitors that do not stabilize DNA-TopoIIα complexes, but instead inhibit ATP hydrolysis, could provide great therapeutic benefit, especially where resistance to conventional TopoIIα poisons is observed.

**Figure 1:**
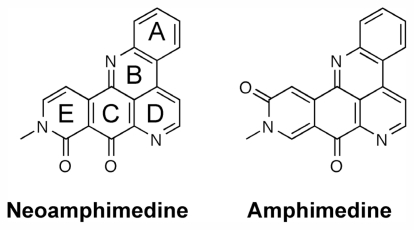
The structures of neoamphimedine and amphimedine.

Neoamphimedine and amphimedine (Figure 1) are marine alkaloids from *Xestospongia* sp. While amphimedine is biologically inactive as an antitumor agent, neoamphimedine is known to inhibit TopoIIα and has been shown to be as effective as etoposide at inhibiting the growth of xenograft tumors in mice [9]. Though neoamphimedines exact mechanism of inhibition is not yet fully understood, it has been demonstrated that neoamphimedine does not stabilize the cleaved complex; rather, it has been shown to induce TopoIIα-mediated catenation of plasmid DNA *in vitro* [9,10].

In the present study we performed TopoIIα-dependent DNA decatenation and cellular proliferation assays with and without neoamphimedine and Metnase to demonstrate that neoamphimedine is able to inhibit Metnase-enhanced TopoIIα activity *in vitro*. TopoIIα-dependent ATP hydrolysis inhibition assays suggest that neoamphimedine is an ATP-competitive inhibitor and *in silico* molecular modeling data supports this indicating that neoamphimedine can bind to the ATPase sites of TopoIIα.

## 2. Results and Discussion

### 2.1. *In Vitro* DNA Decatenation and Cell Proliferation Assays

**Figure 2:**
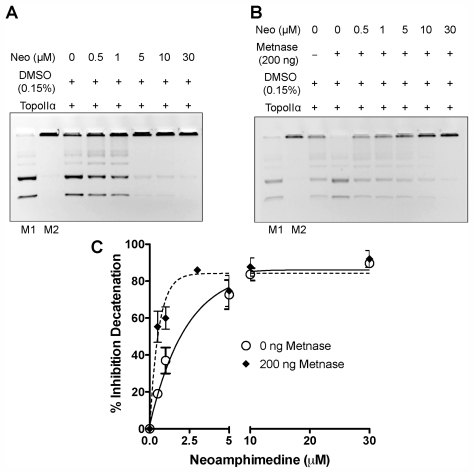
Neoamphimedine inhibits Metnase-enhanced TopoIIα-dependent decatenation. (**a**) A representative TopoIIα-decatenation gel treated with neoamphimedine as indicated; and (**b**) in the presence of Metnase; (**c**) Percent inhibition curves generated from decatenation assays in triplicate. DNA markers are denoted as M1 (decatenated, linear and circular) and M2 (catenated).

Neoamphimedine has been shown to induce DNA catenation *in vitro*; however, the ability of neoamphimedine to inhibit TopoIIα mediated decatenation has not been assessed. In addition, Metnase has been shown to resist the action of TopoIIα poisons by interacting with TopoIIα and enhancing its decatenation activity *in vitro*. Thus, we conducted TopoIIα decatenation assays with neoamphimedine in the presence and absence of an activity-enhancing concentration of Metnase (Figure 2).

Neoamphimedine inhibits TopoIIα-dependent decatenation with and without Metnase over concentrations ranging from 0.5–30 μM. Unexpectedly, neoamphimedines potency increased in the presence of Metnase, suggesting that TopoIIα-Metnase protein-protein interactions may increase the binding affinity of neoamphimedine for TopoIIα (Figure 2C). Previously we reported that the same concentration of Metnase blocks the action of clinically used TopoIIα drugs etoposide and doxorubicin (cleaved complex stabilizers), and ICRF-193 (closed clamp stabilizer) at concentrations that completely inhibit TopoIIα-dependent decatenation in the absence of Metnase [6–8].

**Figure 3:**
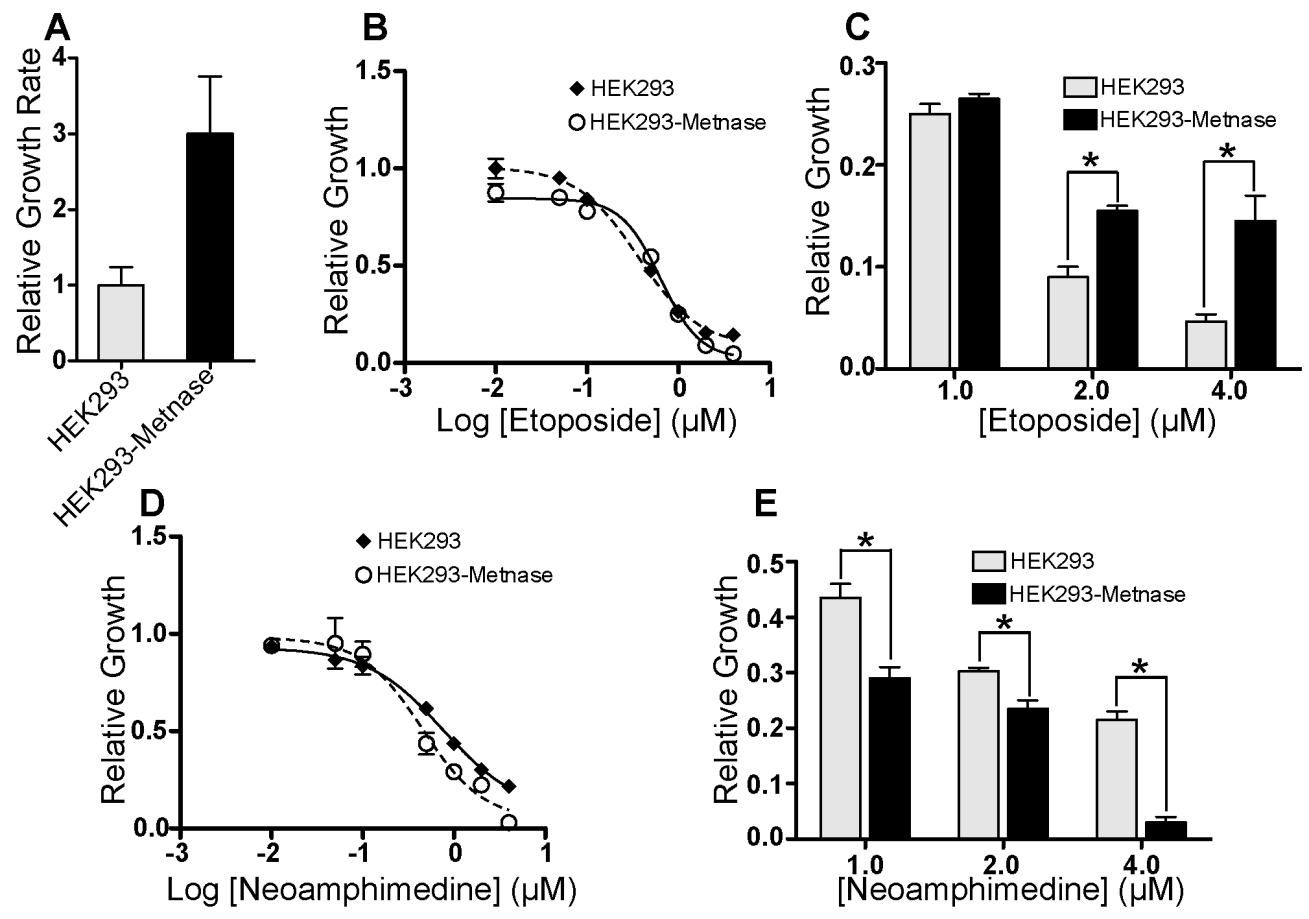
Neoamphimedine inhibits Metnase-enhanced cell proliferation. (**a**) Untreated HEK293 and HEK293-Metnase cell proliferation after 72 hours relative to day 0; (**b**) Relative growth of etoposide treated HEK293 (IC_50_ = 0.6 μM) and HEK293-Metnase (IC_50_ = 0.4 μM) cells to untreated cells after 72 h; (**c**) Relative growth over the concentration range 1–4 μM emphasizing resistance to etoposide in HEK293-Metnase cells; (**d**) Relative growth of neoamphimedine treated HEK293 (IC_50_ = 0.8 μM) and HEK293-Metnase (IC_50_ = 0.5 μM) cells to untreated cells after 72 h; (**e**) Relative growth over the concentration range 1–4 μM emphasizing sensitivity to neoamphimedine in HEK293-Metnase cells. Statistical significance was determined using the unpaired, two-tailed t-test analysis, where (*) denotes *P* ≤ 0.05.

We then investigated the potential for Metnase-related MDR by comparing relative growth of HEK293 cells to HEK293 cells that over-express Metnase (HEK293-Metnase) in the presence of neoamphimedine and etoposide (Figure 3). HEK293-Metnase cells display increased proliferation and resistance to etoposide relative to HEK293 cells as reported (Figure 3B,C) [6,8]. In contrast, over-expression of Metnase increases sensitivity of cells to neoamphimedine, evident at concentrations ≥0.5 μM (Figure 3D,E), consistent with the results observed in our decatenation assays showing increased inhibition of TopoIIα-dependent decatenation with neoamphimedine in the presence of Metnase at concentrations of 0.5 μM or higher (Figure 2C).

### 2.2. Competitive Inhibition Studies with Neoamphimedine and TopoIIα

**Figure 4:**
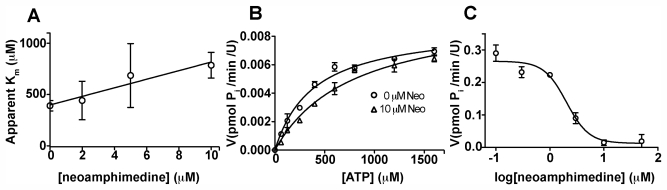
(**a**) A linear (*R*^2^ = 0.94) increase in apparent K_m_ is observed over the range of 0 to 10 μM concentrations of neoamphimedine, indicative of a competitive mode of inhibition; (**b**) Competitive inhibition studies with ATP depicting a shift in the apparent K_m_ from 390 ± 50 to 790 ± 120 μM ATP. Using the unpaired, two-tailed t-test analysis, this shift in apparent K_m_ was statistically significant (*P* = 0.010) while the V_max_ for both curves was not (*P* = 0.186); (**c**) Dose response curve for the inhibition of TopoIIα ATPase function the IC_50_ is found to be 2 μM. All data represent means with SEM error bars.

Since neoamphimedine does not act as a TopoIIα poison it seemed likely that it binds TopoIIα at a different site than classical TopoIIα poisons, which bind near the DNA binding site [2,11,12]. Furthermore, Metnase has also been shown to increase resistance to ICRF-193, a TopoIIα inhibitor that binds allosterically in proximity to the *N*-terminal ATPase site [8,13]. Given that TopoIIα must hydrolyze ATP during catalytic function we hypothesized that Metnase-TopoIIα interactions do not obstruct the *N*-terminal ATPase sites of TopoIIα and that neoamphimedine binds to this site, circumventing Metnase-mediated MDR [14,15]. To test this hypothesis we first measured TopoIIα dependent ATP hydrolysis in the presence of supercoiled DNA, recombinant human TopoIIα and varying concentrations of neoamphimedine using the malachite green assay [16,17]. The results of the ATP hydrolysis studies are displayed in Figure 4. We found the K_m_ of TopoIIα for ATP to be 390 ± 50 μM and V_max_ of the catalysis to be 0.0087 ± 0.0004 pmol min^−1^·U^−1^, consistent with previously reported data [17]. Upon treatment with increasing concentrations of neoamphimedine, apparent K_m_ increased linearly (*R*^2^ = 0.94) while V_max_ did not seem to be dose-responsive, indicative of a competitive mode of inhibition (Figure 4A). Substrate-dependent reaction velocity (Michaelis-Menten plot) is shown in (Figure 4B) for reactions treated with 0 and 10 μM neoamphimedine. The nonlinear fit of the dose-response inhibition model is shown in (Figure 4C). Using 600 μM ATP, the IC_50_ was found to be 2.0 ± 0.4 μM. Using the Cheng-Prusoff equation, the K_i_ was calculated to be 0.8 ± 0.3 μM.

### 2.3. Computational Modeling of Neoamphimedine with TopoIIα

**Figure 5:**
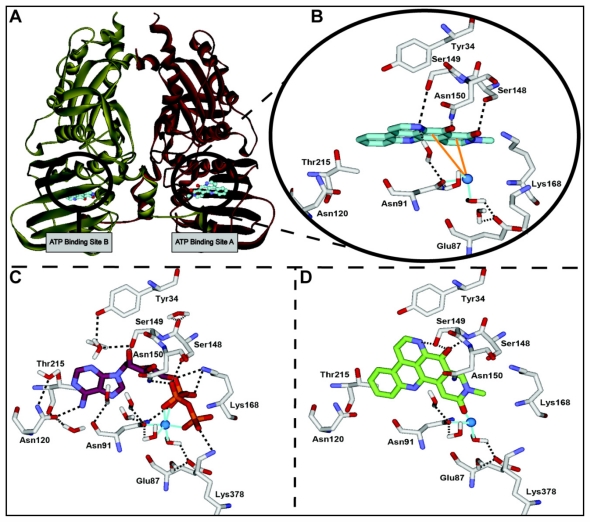
(**a**) The *N*-terminal ATPase domain of TopoIIα; (**b**) Neoamphimedine (cyan carbon atoms) docked in the ATPase binding site with nearby residues (white carbon atoms). Orange lines denote pi-cation attractions. Hydrogen bonds are displayed as black dashes and coordination interactions are shown as light blue lines with Mg^2+^ (blue sphere); (**c**) ATP (burgundy carbon atoms) docked in the ATPase binding site; (**d**) Amphimedine docked (green carbon atoms) in the ATPase binding site of TopoIIα.

To further investigate how neoamphimedine binds and inhibits TopoIIα we conducted computational docking studies. As predicted by our biological studies, the TopoIIα ATPase sites were identified as binding sites of neoamphimedine with favorable calculated binding energies. The results of the computational modeling are shown in Figure 5. The binding conformation of neoamphimedine in the ATPase site of TopoIIα (calculated binding energy = −61.8 kcal/mol) mimics that of ATP, the natural substrate (Figure 5B,C). The key interactions observed with neoamphimedine are a network of hydrogen bonds with Ser148, Ser149, and Asn150, and with Asn91 through an ordered water molecule. Additionally, neoamphimedine is attracted to the ATPase site Mg^2+^ through charge transfer pi-cation interactions. Molecular docking of the inactive isomer amphimedine in the ATPase site of TopoIIα (calculated binding energy = −39.4 kcal/mol) reveals why the position of the carbonyl group is so important (Figure 5D). When the carbonyl is in the 3-position, as in amphimedine, the hydrogen bond with Ser148 is lost. This repositions amphimedine in the ATPase site in a conformation where the ring system is perpendicular compared to the binding of neoamphimedine. As a result of this repositioning, pi-cation interactions observed with neoamphimedine are lost and there is an unfavorable steric interaction with the active site Mg^2+^. Thus, the position of the E ring carbonyl of neoamphimedine is key to its potent biological activity.

### 2.4. Discussion

MDR is a major problem associated with existing clinically used TopoIIα inhibitors [18]. The most common mode of clinical drug resistance is due to the *ABCB1* gene product P-glycoprotein (Pgp) efflux pump [18,19]. Another mode of MDR is associated with alterations in topoisomerase II [19]. One mechanism of MDR involves TopoIIα phosphorylation that results in increased catalytic activity [3,19,20]. Likewise, TopoIIα has been shown to complex with numerous cellular proteins that can alter its function independently of phosphorylation, also leading to increased catalytic activity and MDR in multiple types of cancer [3,7,21]. The most successful TopoIIα inhibitors are poisons that act by stabilizing either the cleaved complex or the closed clamp complex with DNA, which lead to DNA damage and cytotoxicity. Cases of MDR to clinically used TopoIIα poisons are frequently observed, and the nonspecific DNA damage caused by these poisons is known to cause adverse side effects and secondary malignancies. Therefore, inhibitors of TopoIIα that do not stabilize TopoIIα-DNA complexes may have greater therapeutic value than current inhibitors.

Neoamphimedine is a novel TopoIIα inhibitor that does not stabilize the cleaved complex [9]. Nor does it cause significant DNA strand breaks or intercalate into DNA at concentrations below 100 μM [22]. Neoamphimedine is not a substrate for Pgp-dependent MDR, and it has been shown to be as efficacious as current clinical TopoIIα poisons both *in vitro* as an inhibitor of TopoIIα and *in vivo* as an antitumor agent [9]. We show here that neoamphimedine inhibits TopoIIα ATPase function with an IC_50_ of 2 μM and a corresponding K_i_ of 0.8 ± 0.3 μM. Indicative of a competitive mode of inhibition, a linear increase in the apparent K_m_ of TopoIIα for ATP is observed with increasing neoamphimedine concentrations (Figure 4).

Neoamphimedine inhibits TopoIIα-dependent decatenation at low concentrations with 90% inhibition occurring at 30 μM (Figure 2A,C). These data agree with the reported cell based anti-tumor activity of neoamphimedine (IC_50_ values ranging from 1–6 μM) in various human tumor cell lines [9]. In the presence of the TopoIIα enhancer Metnase (Figure 2B,C), neoamphimedine inhibits TopoIIα-dependent decatenation at all concentrations tested (0.5–30 μM). This result is significant because Metnase has been shown to block the action of traditional TopoIIα poisons in both cell-based and *in vitro* decatenation assays, including doxorubicin, etoposide, and ICRF-193, suggesting that neoamphimedine could overcome Metnase-dependent MDR.

Furthermore, in the presence of Metnase the inhibitory activity of neoamphimedine significantly increases in both *in vitro* decatenation assays (Figure 2) and cell based assays (Figure 3), suggesting that TopoIIα-Metnase protein-protein interactions may increase the binding affinity of neoamphimedine for TopoIIα. Neoamphimedine has been shown to induce DNA catenation at concentrations ≥91 μM and saturating concentrations of ATP (1 mM) [9,23]. Our findings indicate that under these conditions ATP would out-compete neoamphimedine for binding to TopoIIα; however, neoamphimedine can induce DNA aggregation at relatively high concentrations (≥91 μM) facilitating catenation in the presence of saturating ATP [9]. ATP hydrolysis has been shown to drive equilibrium to favor decatenation rather than catenation in the absence of DNA aggregation by a condensing agent, and at relatively low concentrations (≤50 μM) neoamphimedine does not induce DNA aggregation [9,24]. Therefore, we propose that the primary anti-tumor activity of neoamphimedine is the inhibition of TopoIIα through binding of the *N*-terminal ATPase sites and competitively inhibiting ATP hydrolysis.

## 3. Experimental Section

### 3.1. General Experimental Procedures

Except where otherwise indicated, all chemicals were purchased from a chemical supplier and used as received. All computational simulations were performed within the Discovery Studio^®^ software [25], and crystallographic coordinates were obtained from the RCSB protein databank [4]. Neoamphimedine preparation [26] and recombinant Metnase purification [27] were performed as previously described.

### 3.2. DNA Decatenation Assay

TopoIIα (TopoGen) was added to 70 ng of kinetoplast DNA (kDNA, TopoGen) comprising small interlocked supercoiled circular DNA in reaction buffer (50 mM Tris-HCl, pH 8.0, 120 mM KCl, 10 mM MgCl_2_, 0.5 mM DTT, 0.5 mM ATP) in the presence or absence of purified recombinant Metnase (200 ng) and various concentrations of neoamphimedine. Reaction mixtures were incubated at 39 °C for an optimized time of 30 min. The reactions were then separated on a 1% agarose gel containing 0.5 μg/mL ethidium bromide with decatenated and catenated DNA markers (TopoGen). Fluorescence intensity quantitative analysis was carried out using Quantity One^®^ software [28] by calculating the percent decatenation by comparing the pixel intensities of both decatenated bands relative to the catenated band. The percent inhibition of decatenation was then determined using the following formula:

$$\%\text{Inhibition Decatenation}=100\left(1-\frac{\%\text{Decatenation with neo}}{\%\text{Decatenation without neo}}\right).$$

### 3.3. Cell Proliferation Assay

HEK-293 cells transfected with a Metnase expression vector (HEK293-Metnase) were described previously [6]. A colorimetric cytotoxicity assay using sulforhodamine B was utilized to compare the proliferative ability of HEK293 and HEK293-Metnase cells [29].

Briefly, cells were cultured in DMEM (Invitrogen, Carlsbad, CA, USA) supplemented with 10% FBS (Sigma, St. Louis, MO, USA), seeded at 2000 cells/100 μL/well in 96-well plates and incubated for 24 h. Medium was then replaced with medium containing various concentrations of neoamphimedine or etoposide. After 72 h treatment, cells were fixed with 10% aqueous trichloroacetic acid for 1 h at 4 °C, and washed 5× with 200 μL water. Dry fixed cells were stained with 0.4% sulforhodamine B in 1% aqueous acetic acid for 30 min, and washed 4× with 200 μL of 1% aqueous acetic acid. Dry stained cells were treated with 10 mM unbuffered Tris-base to extract protein-bound dye and absorbance readings were measured at 535 nm using a microplate reader (EnVision^®^, Perkin Elmer).

### 3.4. Competitive Inhibition Studies

An ATP hydrolysis assay that quantifies inorganic phosphate through the formation of a high molar absorptivity complex with malachite green and molybdate was adapted for use in 384-well plates, with a total reaction volume of 15 μL [16]. We first utilized this assay to determine the K_m_ of TopoIIα for its natural substrate, ATP, using ATP concentrations ranging 62.5–1600 μM. To measure the apparent K_m_, the same reactions were repeated in the presence of 2, 5, or 10 μM neoamphimedine. Absorbance at 620 nm was measured using a SpectraMax^®^ Plus microtiter plate reader and SoftMax^®^ Pro software [30]. To determine the K_i_ of neoamphimedine the same method was used with the following modifications: neoamphimedine (0.1–10 μM) in reaction buffer (without DNA and DTT) was added to the complete reaction buffer and pre-incubated for 10 min at 37 °C. Reactions were initiated by the addition of 600 μM ATP and incubated for 30 min at 37 °C. Reactions were terminated and measured as described. The IC_50_ value was determined and the K_i_ was calculated using the Cheng-Prusoff equation [31]. Experiments were repeated in duplicate and Prism^®^ software [32] was used to perform non-linear fits (method of least squares) to the Michaelis-Menten kinetic model (for K_m_ and V_max_ values) or the dose-response inhibition model (for IC_50_ values).

### 3.5. Molecular Docking of Neoamphimedine and Amphimedine with TopoIIα

The 1.87 Å crystal structure of the ATPase domain of human TopoIIα (PDB: 1ZXM, [4]) was applied using the CHARMm force field [33], and residues were corrected for physiological pH. The binding site was defined as whole residues within an 8 Å radius subset encompassing the ATPase site. LigandFit and Flexible Docking protocols were used for the molecular docking of neoamphimedine into the defined binding site of TopoIIα [34]. Grid resolution was set to 0.5 Å and electrostatic energy was included in the calculation of the ligand internal energy. In order to avoid identical conformations, a root mean square deviation threshold of 1.5 Å and a score threshold of 20 kcal/mol were used. Fifty structural outputs were specified and the identification of a docked conformation was followed by a minimization using the conjugate gradient method to a convergence of 0.001 kcal/mol to optimize ligand-protein interactions. The Poisson-Boltzmann method was used to calculate binding energy, with a non-bonded list radius of 12 Å. The top-ranked conformations for each docked complex were selected on the following criteria: calculated binding energy, and hydrogen bond interactions between the protein and ligand.

## 4. Conclusions

In conclusion, the *in vitro*, cell based, and *in silico* studies presented characterize neoamphimedine as an ATP-competitive inhibitor of TopoIIα. These studies show that neoamphimedine can overcome drug resistance observed with conventional TopoIIα poisons due to TopoIIα-Metnase protein interactions. Molecular modeling has elucidated the pharmacophore of neoamphimedine, most notably the position of the E ring carbonyl and the resulting hydrogen bond with Ser148. Finally, it is reasonable to conclude that Metnase likely binds TopoIIα near the DNA binding site located in the cleavage and relegation domain, which prevents or alters TopoIIα poison binding but does not block access to the *N*-terminal ATPase binding sites of TopoIIα, which is logical given that TopoIIα must hydrolyze ATP during catalytic function [14,15]. These results support the continued development of neoamphimedine as a novel catalytic inhibitor of TopoIIα for the treatment of cancer.

## References

[1] Champoux J.J. (2001). DNA Topoisomerases: Structure, Function and Mechanism. Annu. Rev. Biochem.

[2] Nitiss J.L. (2009). Targeting DNA topoisomerase II in cancer chemotherapy. Nat. Rev.Cancer.

[3] Nitiss J.L. (2009). DNA topoisomerase II and its growing repertoire of biological functions. Nat. Rev. Cancer.

[4] Wei H., Ruthenburg A.J., Bechis S.K., Verdine G.L. (2005). Nucleotide-dependent domain movement in the ATPase domain of a human type IIA DNA topoisomerase. J. Biol. Chem.

[5] Osheroff N., Zechiedrich E.L., Gale K.C. (1991). Catalytic function of DNA topoisomerase II. BioEssays.

[6] Williamson E.A., Rasila K.K., Corwin L.K., Wray J., Beck B.D., Severns V., Mobarak C., Lee S.H., Nickoloff J.A., Hromas R. (2008). The SET and transposase domain protein Metnase enhances chromosome decatenation: Regulation by automethylation. Nucleic Acids Res.

[7] Wray J., Williamson E.A., Royce M., Shaheen M., Beck B.D., Lee S.H., Nickoloff J.A., Hromas R. (2009). Metnase mediates resistance to topoisomerase II inhibitors in breast cancer cells. PLoS One.

[8] Wray J., Williamson E.A., Sheema S., Lee S.H., Libby E., Willman C.L., Nickoloff J.A., Hromas R. (2009). Metnase mediates chromosome decatenation in acute leukemia cells. Blood.

[9] Marshall K.M., Matumoto S.S., Holden J.A., Concepcion G.P., Tasdemir D., Ireland C.M., Barrows L.R. (2003). The anti-neoplastic and novel topoisomerase II-mediated cytotoxicity of neoamphimedine, a marine pyridoacridine. Biochem. Pharmacol.

[10] Guzman F.S.D., Carte B., Troupe N., Faulkner D.J., Harper M.K., Concepcion G.P., Mangalindan G.C., Matsumoto S.S., Barrows L.R., Ireland C.M. (1999). Neoamphimideine: A New Pyridoacridine Topoismerase II Inhibitor Which Cantenates DNA. J. Org. Chem.

[11] Bender R.P., Jablonksy M.J., Shadid M., Romaine I., Dunlap N., Anklin C., Graves D.E., Osheroff N. (2008). Substituents on etoposide that interact with human topoisomerase IIalpha in the binary enzyme-drug complex: contributions to etoposide binding and activity. Biochemistry.

[12] Wu C.C., Li T.K., Farh L., Lin L.Y., Lin T.S., Yu Y.J., Yen T.J., Chiang C.W., Chan N.L. (2011). Structural basis of type II topoisomerase inhibition by the anticancer drug etoposide. Science.

[13] Classen S. (2003). Structure of the topoisomerase II ATPase region and its mechanism of inhibition by the chemotherapeutic agent ICRF-187. Proc. Natl. Acad. Sci. USA.

[14] Baird C., Harkins T., Morris S., Lindsley J. (1999). Topoisomerase II drives DNA transport by hydrolyzing one ATP. Proc. Natl. Acad. Sci. USA.

[15] Maxwell A., Costenaro L., Mitelheiser S., Bates A.D. (2005). Coupling ATP hydrolysis to DNA strand passage in type IIA DNA topoisomerases. Biochem. Soc. Trans.

[16] Lanzetta P.A., Alvarez L.J., Reinach P.S., Candia O.A. (1979). An improved assay for nanomole amounts of inorganic phosphate. Anal. Biochem.

[17] Chene P., Rudloff J., Schoepfer J., Furet P., Meier P., Qian Z., Schlaeppi J.M., Schmitz R., Radimerski T. (2009). Catalytic inhibition of topoisomerase II by a novel rationally designed ATP-competitive purine analogue. BMC Chem. Biol.

[18] Szakacs G., Paterson J.K., Ludwig J.A., Booth-Genthe C., Gottesman M.M. (2006). Targeting multidrug resistance in cancer. Nat. Rev. Drug Discov.

[19] Beck W.T., Danks M.K., Wolverton J.S., Kim R., Chen M. (1993). Drug resistance associated with altered DNA topoisomerase II. Adv. Enzyme Regul.

[20] Matsumoto Y., Kunishio K., Nagao S. (1999). Increased phosphorylation of DNA topoisomerase II in etoposide resistant mutants of human glioma cell line. J. Neurooncol.

[21] Shapiro P.S., Whalen A.M., Tolwinski N.S., Wilsbacher J., Froelich-Ammon S.J., Garcia M., Osheroff N., Ahn N.G. (1999). Extracellular signal-regulated kinase activates topoisomerase IIalpha through a mechanism independent of phosphorylation. Mol. Cell. Biol.

[22] Marshall K.M., Andjelic C.D., Tasdemir D., Concepcion G.P., Ireland C.M., Barrows L.R. (2009). Deoxyamphimedine, a pyridoacridine alkaloid, damages DNA via the production of reactive oxygen species. Mar. Drugs.

[23] Matsumoto S.S., Haughey H.M., Schmehl D.M., Venables D.A., Ireland C.M., Holden J.A., Barrows L.R. (1999). Makaluvamines vary in ability to induce dose-dependent DNA cleavage via topoisomerase II interaction. Anticancer Drugs.

[24] Rybenkov V.V., Ullsperger C., Vologodskii A.V., Cozzarelli N.R. (1997). Simplification of DNA topology below equilibrium values by type II topoisomerases. Science.

[25] (2009). Discovery Studio, version 2.5.5.

[26] LaBarbera D.V., Bugni T.S., Ireland C.M. (2007). The Total Synthesis of Neoamphimedine. J. Org. Chem.

[27] Lee S.H., Oshige M., Durant S.T., Rasila K.K., Williamson E.A., Ramsey H., Kwan L., Nickoloff J.A., Hromas R. (2005). The SET domain protein Metnase mediates foreign DNA integration and links integration to nonhomologous end-joining repair. Proc. Natl. Acad. Sci. USA.

[28] (2009). Quantity One.

[29] Skehan P., Storeng R., Scudiero D., Monks A., McMahon J., Vistica D., Warren J.T., Bokesch H., Kenney S., Boyd M.R. (1990). New colorimetric cytotoxicity assay for anticancer-drug screening. J. Natl. Cancer Inst.

[30] (2004). SoftMax Pro, version 4.8.

[31] Cheng Y., Prusoff W.H. (1973). Relationship between the inhibition constant (*K**_I_*) and the concentration of inhibitor which causes 50 per cent inhibition (*I*_50_) of an enzymatic reaction. Biochem. Pharmacol.

[32] (2007). Prism, version 5.0.3.

[33] Brooks B.R., Brooks C.L., Mackerell A.D., Nilsson L., Petrella R.J., Roux B., Won Y., Archontis G., Bartels C., Boresch S. (2009). CHARMM: The biomolecular simulation program. J. Comput. Chem.

[34] Montes M., Miteva M.A., Villoutreix B.O. (2007). Structure-based virtual ligand screening with LigandFit: pose prediction and enrichment of compound collections. Proteins.

